# Anatomical, biochemical and gene expression studies in four hydrangea cultivars under low temperature stress

**DOI:** 10.3389/fpls.2025.1655585

**Published:** 2026-09-03

**Authors:** Qian Qiao, Chenyu Wang, Jing Li, Kan Liu, Hao Tang, Jiangyong Wang, Yun Gao

**Affiliations:** 1 Shandong Institute of Pomology, Shandong Academy of Agricultural Sciences, Taian, Shandong, China; 2 Science and Technology Development Center of the Ministry of Agriculture and Rural Affairs, Beijing, China; 3 College of Plant Protection, Shandong Agricultural University, Taian, Shandong, China

**Keywords:** hydrangea, cold resistance, LT50, membership function, antioxidant enzyme, gene expression

## Abstract

*Hydrangea*, which has large and vivid flowers, is a crucial ornamental plant that is used in landscape design. However, its limited tolerance to cold restricts its widespread application in regions with low temperatures. To investigate the effects of low-temperature stress on hydrangea, four common cultivars were examined. By comparing leaf anatomical structures and physiological parameters including soluble substances levels, antioxidant enzyme activities, and cold resistance-related gene expression levels under different low-temperature conditions (4°C, 0°C, -4°C, -8°C, and -12°C), cold resistance was comprehensively evaluated. The semilethal temperature (LT50) values of the four cultivars ranged from -2.404°C to -3.133°C, and ‘Magic coral’ and ‘Forever summer’ had the lowest values. Under low-temperature stress, the malondialdehyde (MDA) production rate in hydrangea leaves significantly increased, whereas the antioxidant enzymes activity and the soluble substances contents initially increased but subsequently decreased with decreasing temperature. Moderate low-temperature stress increased the expression of antioxidant enzymes and cold resistance-related genes (*ERF*, *POD*, *CHS*, and *ANS*), thus increasing the activity of intracellular antioxidant defense systems. This mechanism contributed to enhancing cell membrane stability and increasing overall cold resistance in hydrangeas. A comprehensive evaluation was conducted using the membership function method, which allowed the allowing for the analysis of both leaf anatomical structure indices and physiological indicators. The results revealed the cold resistance of the four selected hydrangea cultivars, indicating that the cold tolerance of ‘Forever summer’ and ‘Magic coral’ was superior to that of ‘Tabei’ and ‘Huabao’. Therefore, ‘Forever summer’ and ‘Magic coral’ are recommended for use as priority cultivars for in landscape applications in regions with low temperatures.

## 1 Introduction

*Hydrangea*, which is a genus within the family Saxifragaceae, comprises primarily shrubs and subshrubs, and a minority of its members are small trees (Li et al., 2020; Yuan et al., 2023). The hydrangea has a cymose inflorescence, which has a spherical or ovoid shape, and the entire inflorescence is clustered among the oval-shaped leaves; thus, this plant has extremely high ornamental value (Chen et al., 2025; Gong et al., 2024). Moreover, the flowers of hydrangea have a pleasant fragrance and a long flowering period. Written records of the application of hydrangeas in courtyard planting is found in records as early as ancient Chinese flower monographs. In addition to its use in solitary planting and cluster planting, hydrangea can also be used as the main element of flower beds, and it is used in courtyards and landscaping (Ahmad et al., 2024; Wang et al., 2022). To date, more than 600 cultivars of hydrangea have been cultivated, and hydrangea is among the most economically valuable ornamental plants in the United States (Alexander, 2017). Furthermore, hydrangea has recently emerged as a cut flower commodity that grows rapidly. Statistical data indicate that hydrangea has become the sixth most popular species of cut flower after roses, chrysanthemums, carnations, gladioli, and gerberas, demonstrating significant market expansion (Nordli et al., 2011).

Hydrangeas should be planted in zones with temperatures ranging from 18~28°C as they prefer shade, moisture, and warmth. Notably, hydrangeas are not resistant to cold. Indeed, low-temperature freezing damage often occurs in early spring and late autumn in northern China (Feng et al., 2024; Pagter et al., 2008), and this damage restricts the promotion and application of hydrangeas in that region. Studies have shown that when plants are subjected to cold-induced damage stress, they produce large amounts of free radicals and reactive oxygen species (ROS), which cause membrane lipid peroxidation, reduce or decrease protein activity, and lead to metabolic disorders in plants (Amini et al., 2021; Liu et al., 2023; Ying et al., 2025). If plants are exposed to low-temperature stress for a long period, their physiological and biochemical characteristics, such as membrane phase, osmotic regulatory substances accumulation, and variations in the oxidase system, change (Cao et al., 2017; Qian et al., 2024). Therefore, cold resistance is an important factor that must be considered in the production and application of hydrangeas. Therefore, in this study, four common cultivars of hydrangea were selected as experimental materials. By comparing leaf anatomical structure indices and parameters, such as osmotic adjustment substances levels, antioxidant enzyme activities, and cold resistance-related gene expression, under different low-temperature conditions, the degree of cold resistance in these cultivars were evaluated. A comprehensive assessment and screening were conducted to provide a theoretical basis for the introduction, domestication, and landscape application of hydrangeas in northern regions with cold climates.

## 2 Materials and methods

### 2.1 Test materials

In this study, two-year-old cut sapling of four hydrangea cultivars namely, ‘Magic coral’, ‘Forever summer’, ‘Tabei’, and ‘Huabao’, were used as the experimental materials. These four cultivars were obtained from the Potted Flower Research Institute of Dafeng City, Jiangsu Province. In spring (May), 45 healthy, uniformly growing, and pest-free cuttings of each cultivar were selected and transplanted to pots (14*9.2*12 cm). The plants were cultivated in a greenhouse under controlled conditions (temperature: 25°C; humidity: 65%; light intensity: 200 μE·m^-^²·s^-^¹; photoperiod: 14 h light/10 h dark) for two weeks. The experiment included five treatments, with three plants per treatment group and three replicates. A temperature of 4°C was used as the control, and the plants were subjected to low-temperature stress at 0°C, -4°C, -8°C, or -12°C for 2 h in an artificial climate chamber. From each cultivar and treatment group, the 2nd–3rd mature leaves from the main branches of the current year were collected and used to analyze electrical conductivity and determine of various related physiological indicators. Additionally, a portion of the leaves was immediately frozen in liquid nitrogen and stored at -80°C, and this portion was used for RNA extraction and quantitative real-time polymerase chain reaction (qRT-PCR) analysis. The entire experiment was repeated three times to ensure the reproducibility of the results.

### 2.2 Test methods

#### 2.2.1 Observation of leaf anatomical structure

Leaves from the third leaf position from the top to the bottom of each cultivar that had not been subjected to low-temperature conditions (25°C) were collected, and the midrib part of the leaf was removed. The samples were fixed in a formalin-acetic acid-alcohol (FAA) solution and then processed via dehydration, clearing, and embedding procedures. Sections with a thickness of 8 μm were prepared, double-stained with safranin-fast green, mounted with neutral balsam, and dried. The prepared paraffin sections were observed and photographed with a Leica fluorescence inverted microscope (DMLB, Leica, Germany). The thicknesses of the leaf, upper epidermis, lower epidermis, cuticle, palisade tissue, and spongy tissue were measured with Leica Application Suite image analysis software. The palisade to sponge tissue ratio, mesophyll tissue compactness, and mesophyll tissue porosity were subsequently calculated. Notably, the measured data are presented as the average values of five visual fields.

#### 2.2.2 Method for determining the physiological indices of cold resistance

The relative electrical conductivity (REC) was determined using the conductivity method. Leaf samples (avoiding midribs and margins) were cut into 5mm×5mm pieces, and a 0.2g sample was weighed and placed in test tubes with 20 mL of deionized water. After the test tube was closed, it was placed in a constant-temperature oscillation box and fully oscillated for 1 h, and the initial conductivity (S1) was measured with a conductivity meter (DDSJ-308A, Lei-ci, Shanghai, China). The tubes were then incubated in a boiling water bath for 15 min and cooled to room temperature, after which the final conductivity (S2) was measured. The experiment was repeated three times.

Samples from each group were cut and mixed evenly under different temperature gradients, and 0.5 g of leaf tissue was accurately placed in a precooled mortar. Small amounts of quartz sand and liquid nitrogen were added to promote thorough homogenization, and the mixture was transferred to a centrifuge tube with 5 mL of a phosphate buffer solution (50 mmol/L, pH 7.8). The centrifuge tube was placed in a freezing centrifuge at 4°C and centrifuged at 10000 rpm/min for 20 min. The resulting supernatant (enzyme extract) was subjected to physiological assays. The soluble protein content was measured via the Coomassie brilliant blue method, the soluble sugar content was determined via the anthrone colorimetric method, the superoxide dismutase (SOD) activity was determined via the nitroblue tetrazolium method, the peroxidase (POD) activity was calculated via the guaiacol method, the catalase (CAT) activity was measured via the ammonium molybdate colorimetric method, and the malondialdehyde (MDA) content was determined via the thiobarbituric acid method (Gao et al., 2024). This process was repeated 3 times, and the average values were calculated and analyzed statistically.

#### 2.2.3 Gene expression analysis

Total RNA was extracted from hydrangea leaves with a TRIzol reagent kit (Invitrogen, Thermo Fisher Scientific, Braunschweig, Germany), the RNA concentration and purity were determined via Nanodrop 2000 spectrophotometry (Thermo Fisher Scientific, Waltham, MA, USA), and the integrity was verified by 1% agarose gel electrophoresis. cDNA synthesis was performed following the cDNA synthesis kit protocol (Fermentas, Waltham, MA, USA). qRT-PCR was conducted with an ABI7500 system with TaKara SYBR Premix Ex Taq according to the manufacturer’s instructions (Takara, Dalian, China). The amplification program included the following: initial denaturation at 95°C for 30 s, followed by 40 cycles of 95°C for 5 s and 52-55°C for 20 s. Three technical replicates were included for each treatment. Gene-specific primers (F/R) were designed, and *β-TUB* was used as the reference gene (Chen et al., 2022); the primers were synthesized according to the description provided by Hu et al. (2016) (**Table 1**). Relative gene expression levels under different conditions were calculated via the 2^-ΔΔCT^ method on the basis of the obtained CT values.

**Table 1:** Gene-specific primers that were used in the experiment.

Gene name	Primer sequence
*CHS*	F: ACCGTCGAGGAAGTCCGTAAGG
	L: AATTAGGTGGCGTCGCAGTTCC
*ANS*	F: GCCACGAGCGAGTATGCGAAG
	L: CCAGCCTTCCTTCTTCCAAGCC
*ERF5*	F: CGTGGAAGCAAGGCGATAC
	R: ACGGCGTCAAAGGACAAA
*POD47*	F: GAAATGGTGGCTTTGTCCG
	R: AATGGTTGCTCCGTGTTGT
*β-TUB*	R: ATGAGCACCAAGGAAGTCGAC
	F: CTGAACATCTCTTGAATCGAGGTC

### 2.3 Data analysis and processing

The semilethal temperature (LT50) of each plant was determined by fitting a logistic regression equation (y = k/(1 + ae^(-bx)) to the REC data, where k, a, and b are constants, following the methodology described by Jin et al. (2022).

SPSS 22.0 software was used to assess the statistical significance of differences in various indicator levels. The membership function method (Gholizadeh et al., 2022; Zhang et al., 2025) was to comprehensively evaluate cold resistance, in which the membership values of all the indices were summed and averaged. For physiological parameters that were positively correlated with cold resistance, the membership value was calculated as R(Xi) = (Xi-Xmin)/(Xmax-Xmin). For parameters that were negatively correlated with cold resistance, the inverse membership function was applied, i.e., R(Xi) = 1-(Xi-Xmin)/(Xmax-Xmin). The four hydrangea cultivars were then ranked on the basis of their mean membership function values, in descending order, to comprehensively evaluate their cold resistance characteristics.

## 3 Results and analysis

### 3.1 Leaf anatomical structure indices and multiple comparisons of the four hydrangea cultivars under low-temperature stress conditions

**Figure 1:**
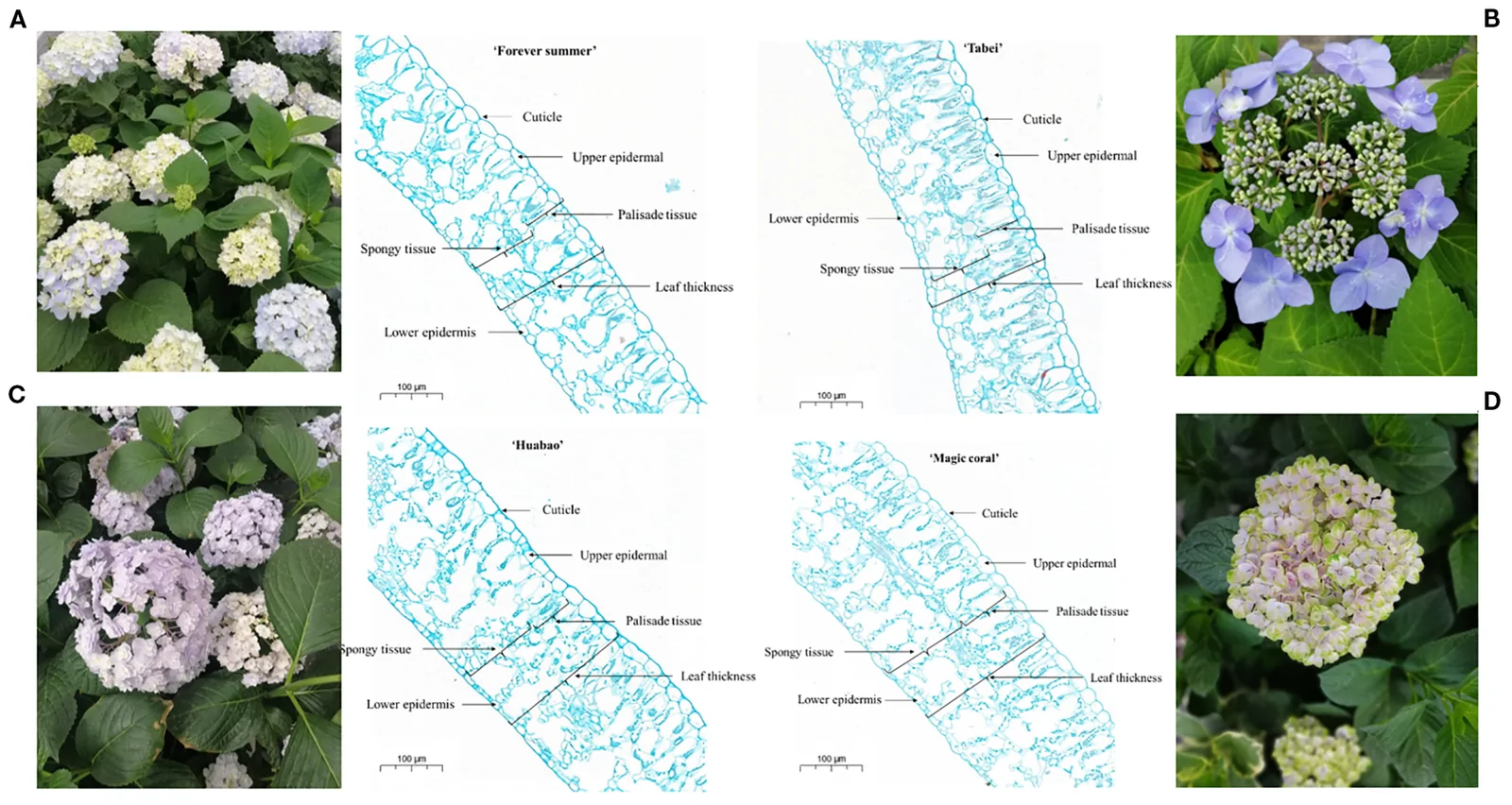
Anatomical structures of the leaves of four hydrangea cultivars. **(A)** ‘Forever summer’; **(B)** ‘Tabei’; **(C)** ‘Huabao’; **(D)** ‘Magic coral’.

The outermost layer of plant leaves consists of a cuticular membrane comprising lipid-like substances, and these substances are the primary protective barrier against various environmental stresses. This cuticle covers the outer cell walls of the leaf epidermis. In generally, a thicker cuticle provides greater protection. However, our measurements (**Table 2**, **Figure 1**) revealed no significant differences in cuticle thickness among the four hydrangea cultivars, indicating that this characteristic does not contribute to the variation in cold tolerance.

**Table 2:** Leaf anatomical structure indices and membership function values of the 4 hydrangea cultivars.

Indicators	‘Forever summer’	‘Tabei’	‘Huabao’	‘Magic coral’	Average	CV/%
Leaf thickness/um	0.3279 ± 0.0015 B	0.3051 ± 0.0143 C	0.2522 ± 0.0084 D	0.3618 ± 0.0058 A	0.3117	13.38
Cuticle/um	0.0026 ± 0.0005	0.0025 ± 0.0004	0.0025 ± 0.0004	0.0025 ± 0.0006	0.0025	17.49
Upper epidermal/um	0.0276 ± 0.0018	0.0256 ± 0.005	0.0207 ± 0.0056	0.0272 ± 0.0018	0.0253	18.16
Lower epidermal/um	0.0129 ± 0.0010 C	0.0160 ± 0.0023 AB	0.0137 ± 0.0014 BC	0.0176 ± 0.0021 A	0.0151	16.58
Palisade tissue/um	0.0960 ± 0.0210 A	0.0872 ± 0.0067 A	0.0664 ± 0.0046 B	0.0836 ± 0.0063 A	0.0833	18.45
Sponge tissue/um	0.1868 ± 0.0219 B	0.1716 ± 0.0170 B	0.1465 ± 0.0084 C	0.2306 ± 0.0113 A	0.1839	18.74
Palisade to sponge tissue ratio	0.5287 ± 0.1605	0.5133 ± 0.0795	0.4546 ± 0.0402	0.3643 ± 0.0417	0.4652	23.39
Mesophyll tissue compactness	0.2925 ± 0.0631	0.2862 ± 0.0268	0.2636 ± 0.0198	0.2313 ± 0.0192	0.2684	15.6
Mesophyll tissue porosity	0.5700 ± 0.0693 b	0.5616 ± 0.0333 b	0.5806 ± 0.0161 b	0.6371 ± 0.0245 a	0.5873	8.24
Average value of the membership function values	0.5228	0.4818	0.2702	0.5086	–	–
Order	1	3	4	2	–	–

Numbers represent mean values ± standard deviations (SDs). Different capital letters in the same row indicate extremely significant differences among cultivars, whereas different lowercase letters represent significant differences, as determined by ANOVA using the Duncan multiple comparison method.

The leaf epidermis, which is a specialized protective tissue comprising tightly arranged parenchyma cells, forms the surface layer of plant leaves. A thicker epidermis typically increases the resistance of plants to damage. Our multiple comparisons of epidermal and mesophyll anatomical characteristics among the four hydrangea cultivars (**Table 2**, **Figure 1**) revealed that the average leaf thickness of the four types of hydrangeas was 0.3117 μm, with a thickness ranking of ‘Magic coral’>‘Forever summer’>‘Tabei’> ‘Huabao’ and significant differences among the cultivars.

The mesophyll tissue of hydrangea flowers is differentiated into two parts, with tighter palisade tissue on the upper front side and looser sponge tissue on the lower back side (**Figure 1**). In general, the higher the aspect ratio of a leaf is, the more developed the palisade tissue is, and the tighter the leaf tissue structure is, resulting in greater protective ability. The thickness of the fence tissue was measured, and the results indicated the following order: ‘Forever summer’>‘Tabei’>‘Magic coral’>‘Huabao’. There were significant differences among ‘Forever summer’, ‘Tabei’, ‘Magic coral’ and ‘Huabao’. The sponge tissue thickness values followed the order of ‘Magic coral’>‘Forever summer’>‘Tabei’>‘Huabao’. The palisade to sponge tissue ratio followed the order of ‘Forever summer’>‘Tabei’>‘Huabao’>‘Magic coral’. There was no significant difference in the mesophyll tissue compactness among the different cultivars, whereas a significant difference in the mesophyll tissue porosity was noted among the different cultivars. ‘Magic coral’ exhibited the greatest difference, which was significantly different from those of ‘Forever summer’, ‘Tabei’ and ‘Huabao’.

Furthermore, the membership function method was used to comprehensively evaluate the correlations between the 9 indicators of leaf anatomical structure and cold resistance (**Table 2**). The ranking of the 4 hydrangea cultivars was ‘Forever summer’ > ‘Magic coral’ > ‘Tabei’ > ‘Huabao’.

### 3.2 Effects of low-temperature stress on the membrane permeability of leaf cells in the four hydrangea cultivars

**Figure 2:**
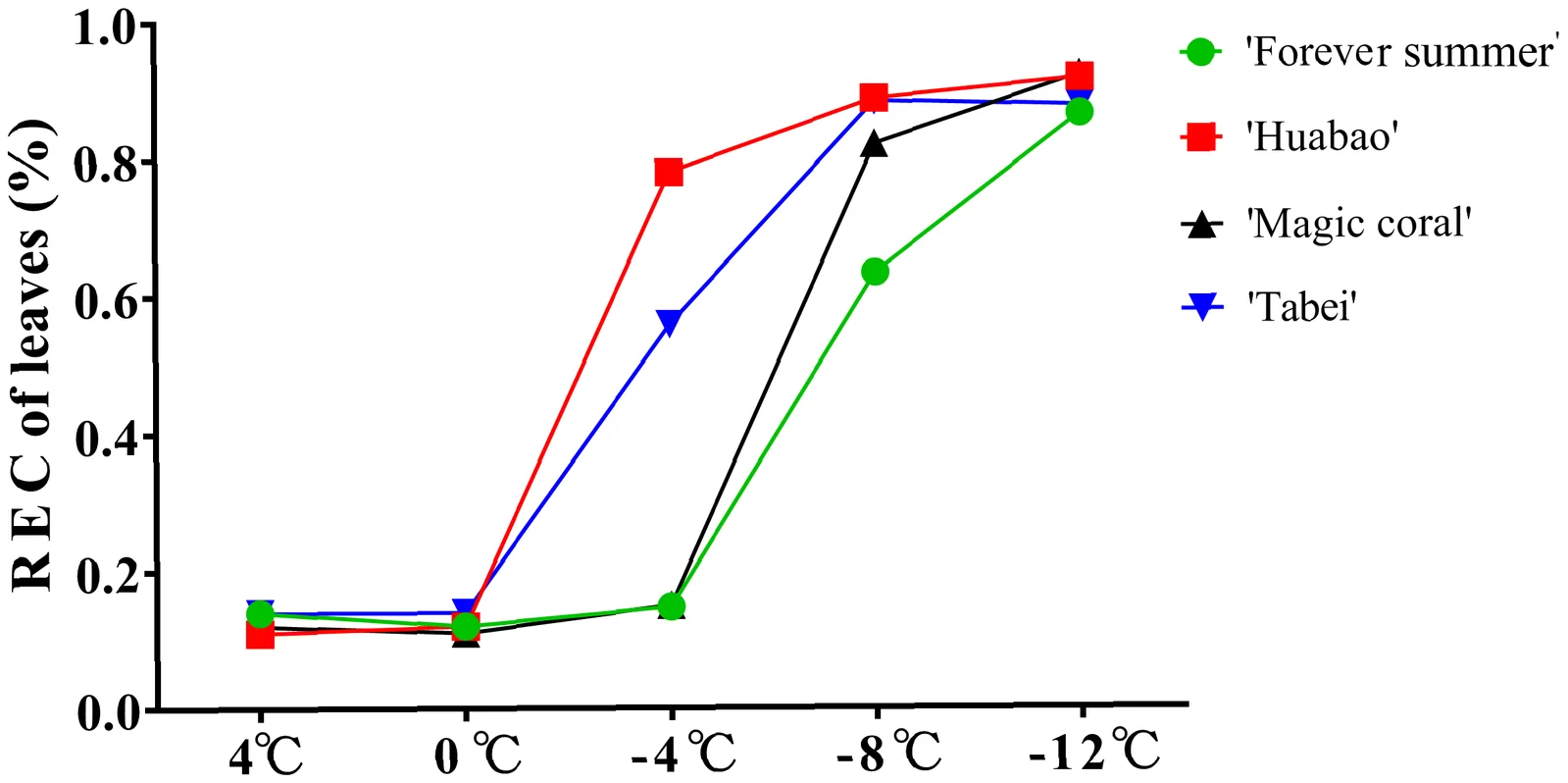
Relative conductivity of the four hydrangea cultivars under low-temperature stress conditions.

As the stress temperature decreased, the REC values of the leaves from all four hydrangea cultivars progressively increased (**Figure 2**). When the temperature decreased from 0°C to -4°C, the REC value of ‘Huabao’ leaves sharply increased and was significantly greater than that of the other three cultivars; these results indicated that the cell membranes of ‘Huabao’ were already damaged at -4°C, and they exhibited significantly greater damage than those of the other cultivars. Throughout the low-temperature stress process, both ‘Magic coral’ and ‘Forever summer’ maintained relatively low REC values, indicating relatively high cold resistance.

Furthermore, a logistic equation was constructed on the basis of the REC values, and the LT50 values were calculated (**Table 3**); that results revealed LT50 values of -3.133°C for ‘Magic coral’, -2.757°C for ‘Forever summer’, -2.711°C for ‘Tabei’, and -2.404°C for ‘Huabao’. On the basis of LT50 analysis, the cold resistance of the four hydrangea cultivars followed the order of ‘Magic coral’>‘Forever summer’>‘Tabei’>‘Huabao’.

**Table 3:** Logistic regression equation and LT50 values of the 4 hydrangeas cultivars.

Cultivars	Logistic equation	LT50/°C	Goodness of fit (R^2^)
‘Magic coral’	y=100/(1 + 56.340e^-1.287x^)	-3.133	0.885
‘Huabao’	y=100/(1 + 76.946e^-1.807x^)	-2.404	0.938
‘Forever summer’	y=100/(1 + 207.058e^-1.934x^)	-2.757	0.901
‘Tabei’	y=100/(1 + 31.855e^-1.277x^)	-2.711	0.892

### 3.3 Physiological indices of the four hydrangea cultivars under low-temperature stress

#### 3.3.1 Effects of low-temperature stress on the MDA content of the 4 hydrangea cultivars

**Figure 3:**
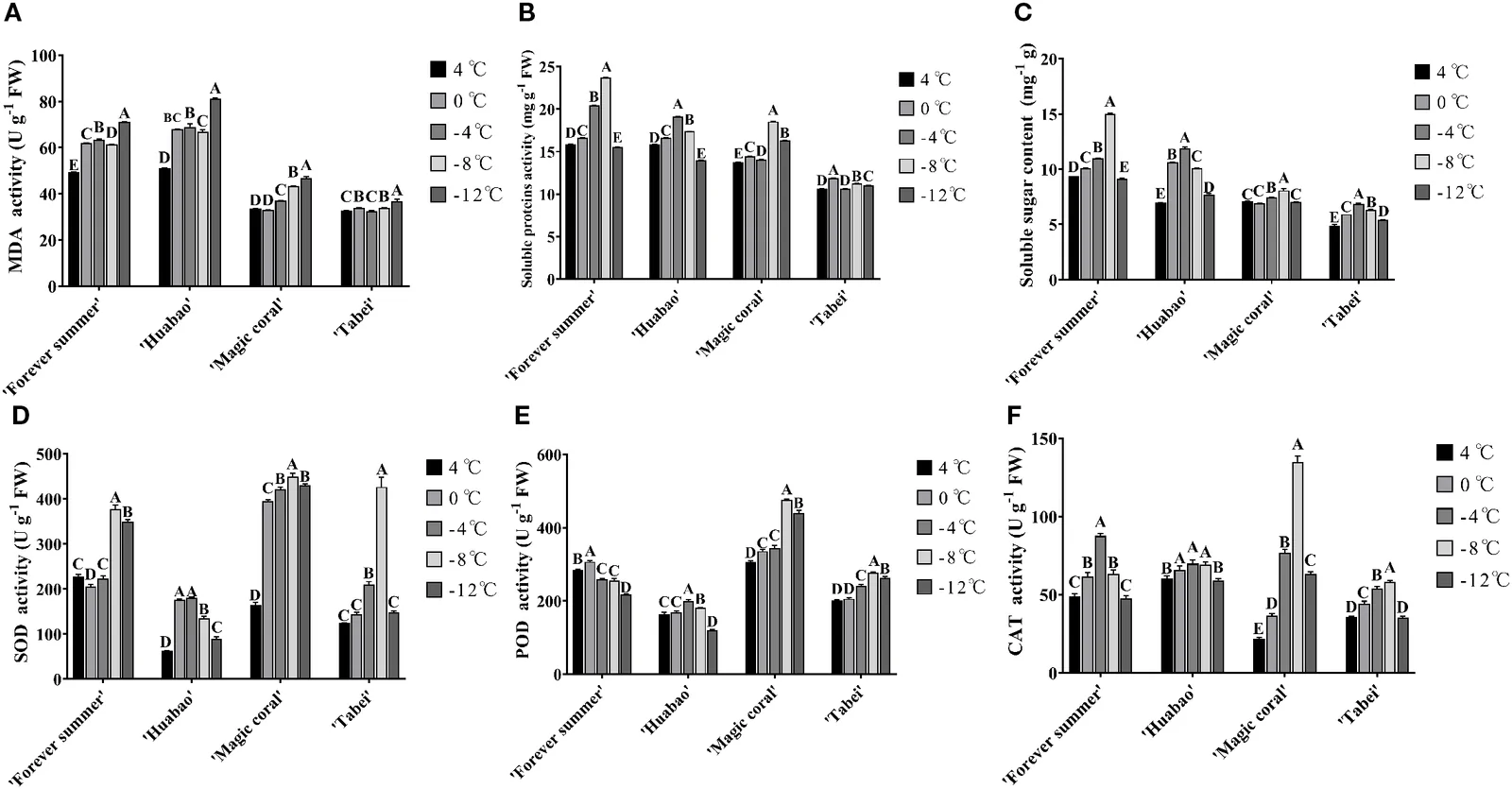
Physiological indices of four hydrangea cultivars under low-temperature stress. **(A)** MDA; **(B)** soluble protein; **(C)** soluble sugar; **(D)** SOD; **(E)** POD; **(F)** CAT. Different capital letters indicate extremely significant differences among cultivars as determined by ANOVA using the Duncan multiple comparison method.

The MDA contents in the four hydrangea cultivars increased with decreasing temperature, with ‘Forever summer’ and ‘Huabao’ demonstrating obvious trends, whereas ‘Magic coral’ and ‘Tabei’ demonstrated gradual trends. However, the changes were not obvious and peaked at -12°C. The MDA contents of ‘Forever summer’ and ‘Huabao’ were significantly greater than those of ‘Magic coral’ and ‘Tabei’, and the maximum average difference was nearly two times greater (**Figure 3A**).

#### 3.3.2 Effects of low-temperature stress on the soluble protein and soluble sugar contents of the 4 hydrangea cultivars

Soluble proteins are crucial osmoregulatory substances and nutrients, and they are often used as indicators for assessing resistance. As shown in **Figure 3B**, the soluble protein content in all four hydrangea cultivars initially increased but then decreased with decreasing temperature. ‘Forever summer’ exhibited the most notable trend, and its soluble protein content peaked at -8°C. When the temperature decreased from 0°C to -4°C, ‘Huabao’ was the first cultivar to exhibit an increase in soluble protein content, indicating rapid induction of protein synthesis to combat cold stress. From -4°C to -8°C, ‘Magic coral’, ‘Forever summer’ and ‘Tabei’ continued to accumulate soluble proteins, whereas the soluble protein content in ‘Huabao’ decreased, suggesting that its protein-synthesizing enzyme systems were already impaired at this stage. Overall, the maximum soluble protein content was observed in ‘Forever summer’, followed by ‘Huabao’, ‘Magic coral’ and ‘Tabei’.

Changes in soluble sugar content serve as important indicators of plant responses to environmental stress. As shown in **Figure 3C**, the soluble sugar content in all four hydrangea cultivars generally initially increased but then decreased with decreasing temperature. When the temperature decreased from 4°C to -4°C, all the cultivars presented increasing soluble sugar levels, demonstrating that these levels increased under cold stress as a protective mechanism. However, from -4°C to -8°C, the soluble sugar levels in ‘Forever summer’ and ‘Huabao’ continued to increase, whereas the soluble sugar levels in ‘Magic coral’ and ‘Tabei’ began to decrease, indicating that the latter two cultivars exhibited more severe vulnerability to cold stress. Overall, the average soluble sugar content was greater in ‘Forever summer’ and ‘Huabao’ than in ‘Magic coral’ and ‘Tabei’.

#### 3.3.3 Effects of low-temperature stress on the activity of antioxidant enzymes in the 4 hydrangea cultivars

Antioxidant enzymes play crucial roles in scavenging the ROS that are induced by environmental stress (Wu et al., 2024). Three key enzymes, i.e., CAT, POD, and SOD, are ubiquitous in plant cells and function as intracellular ROS sensors. These enzymes regulate ROS levels via direct scavenging, converting ROS into less reactive and less toxic compounds.

SOD activity is an important indicator of plant resistance to stress. Higher SOD activity indicates greater antioxidant capacity. As shown in **Figure 3D**, as the stress temperature decreased, the SOD activity in the four hydrangea cultivars generally initially increased but then decreased. Among the cultivars, ‘Huabao’ exhibited peak SOD activity at -4°C, whereas the SOD activity level in the other three cultivars continued to increase, peaking at -8°C; these results indicated that at -4°C, the low-temperature tolerance limit of ‘Huabao’ was exceeded. The SOD levels varied significantly among the four cultivars, with ‘Magic coral’ exhibiting the highest value, followed by ‘Forever summer’ and ‘Tabei’, and ‘Huabao’ exhibiting the lowest value, which was only 34.64% of that of ‘Magic coral’. A comparison of the SOD activity among the four hydrangea cultivars revealed that the cold resistance of ‘Magic coral’ was greater than that of ‘Forever summer’, which was greater than that of ‘Tabei’, with ‘Huabao’ exhibiting the lowest cold resistance.

POD is present in a wide variety of plants, and it is a highly active enzyme. POD can reduce the level of hydrogen peroxide, which can damage plant cells and accelerate senescence; thus, POD protects plants from harm and delays aging. As shown in **Figure 3E**, as the stress temperature decreased, the POD activity in the four hydrangea cultivars generally initially increased but then decreased, indicating that POD began to function in resistance low-temperature stress. Among the cultivars, ‘Magic coral’ showed the most significant change in POD activity, which peaked at -8°C, whereas the other three cultivars presented relatively minor fluctuations: the POD activity in ‘Forever summer’ peaked at 0°C, that in ‘Huabao’ peaked at -4°C, and that in ‘Tabei’ peaked at -8°C. The POD levels significantly differed among the four cultivars, with ‘Magic coral’ demonstrating the highest average value, followed by ‘Forever summer’, ‘Tabei’, and ‘Huabao’. The highest POD activity in ‘Magic coral’ was approximately more than twice that in ‘Huabao’. On the basis of POD activity alone, the cold resistance of the four cultivars can be ranked as follows: ‘Magic coral’ > ‘Forever summer’ > ‘Tabei’ > ‘Huabao’.

After the four hydrangea cultivars were subjected to low-temperature stress, their CAT activity first increased and then decreased (**Figure 3F**), with the greatest change occurring in ‘Magic coral’. The CAT activity in ‘Forever summer’ and ‘Huabao’ peaked at -4°C, whereas that in ‘Magic coral’ and ‘Tabei’ peaked at -8°C. Among the four cultivars, the CAT activity in ‘Tabei’ was the lowest, and the average values of the other three samples did not differ notably. On the basis of the CAT activity, the cold resistance of the four cultivars decreased in the following order: ‘Magic coral’ > ‘Forever summer’ > ‘Huabao’ > ‘Tabei’.

### 3.4 Comprehensive physiological evaluation of the cold resistance in the four hydrangea cultivars

In this study, the membership function method was used to quantitatively transform the seven physiological indicators in the four hydrangea cultivars, yielding average membership values for each indicator in each cultivar. A higher average value indicates greater cold resistance. As indicated in **Table 4**, under all the low-temperature stress treatment conditions, the average membership values of the four hydrangea cultivars varied between 0.379 and 0.534. On the basis of these average values, the order of cold resistance was as follows: ‘Forever summer’ > ‘Magic coral’ > ‘Huabao’ > ‘Tabei’. Here, four physiological indicators, namely, soluble protein content, soluble sugar content, SOD activity, and POD activity, play major roles in the cold resistance of ‘Forever summer’, whereas four physiological indicators, namely, MDA content, SOD activity, POD activity, and CAT activity, are mainly responsible for enhancing the cold resistance of ‘Magic coral’.

**Table 4:** Comprehensive evaluation of the average membership degrees of various physiological indicators and cold resistance of the four hydrangeas cultivars.

Cultivars	REC value	MDA	Soluble protein content	Soluble sugar content	SOD activity	POD activity	CAT activity	Average	Order
‘Forever summer’	0.373	0.590	0.777	0.597	0.543	0.406	0.346	0.519	1
‘Huabao’	0.534	0.708	0.700	0.455	0.170	0.133	0.372	0.439	3
‘Magic coral’	0.322	0.127	0.651	0.251	0.783	0.730	0.387	0.464	2
‘Tabei’	0.483	0.030	0.467	0.111	0.376	0.331	0.208	0.287	4

The data on 7 physiological and biochemical characteristics included the values obtained over the entire temperature range included in these experiments, i.e., 4, 0, -4, -8, and -12°C.

### 3.5 Analysis of the differential expression of cold resistance-related genes

**Figure 4:**
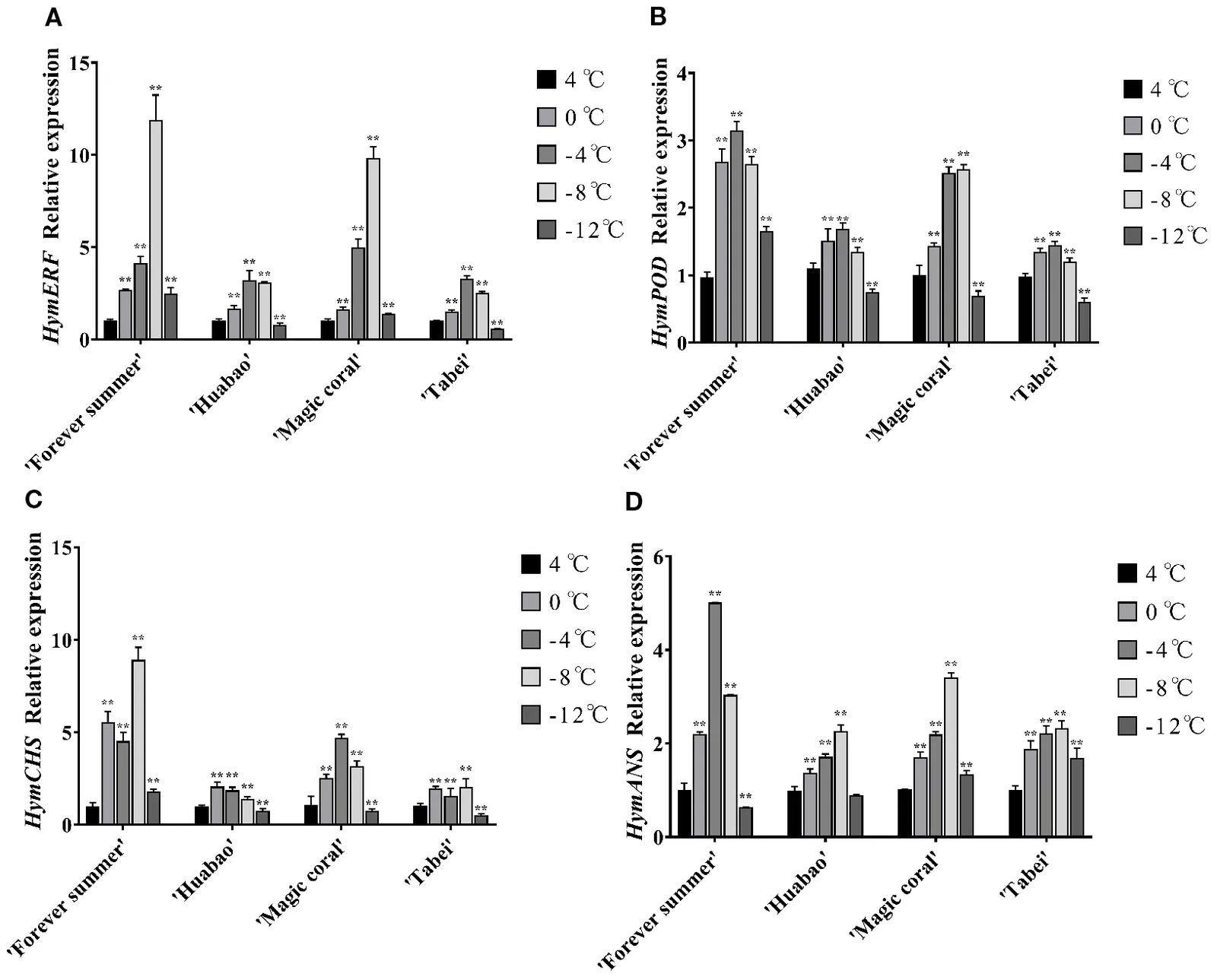
Analysis of the differences in the expression of four genes under different low-temperature conditions. **(A)** *HymERF*; **(B)** *HymPOD*; **(C)** *HymCHS*; **(D)** *HymANS*. ** indicates extremely significant differences among cultivars as determined by ANOVA using the Duncan multiple comparison method.

When plants are exposed to low temperatures, they induce the expression of a series of functional genes that are related to low-temperature stress through the regulation of gene transcription by transcription factors; this mechanism allows plants to increase their overall resistance to low temperatures (Ashraf, 2010; Lee et al., 2005). As shown in **Figure 4**, under continuous low-temperature stress conditions, the expression levels of four cold resistance-related genes in the four hydrangea cultivars first increased and then decreased. The expression of the transcription factor *HymERF* differed most significantly with temperature changes in ‘Forever summer’ and ‘Magic coral’, and in both cultivars, *HymERF* expression peaked at -8°C. However, the differences in expression between ‘Huabao’ and ‘Tabei’ were relatively small, and in these cultivars, *HymERF* expression peaked at -4°C was significantly lower than that in ‘Forever summer’ and ‘Magic coral’ (**Figure 4A**). *HymPOD*, which is an antioxidant enzyme-encoding gene, exhibited the greatest increase in expression in ‘Forever summer’, significantly surpassing that in ‘Magic coral’, ‘Huabao’, and ‘Tabei’. *HymPOD* expression peaked at -4°C and sharply decreased with further decreases in temperature. When the temperature decreased to -12°C, the expression levels in ‘Magic coral’, ‘Huabao’, and ‘Tabei’ were all below those at 4°C (**Figure 4B**). The expression levels of *HymCHS* and *HymANS*, which are two structural genes that are involved in flavonoid and anthocyanin biosynthesis, were also strongly influenced by low temperature. *HymCHS* exhibited the greatest change in expression in ‘Forever summer’, peaking at -8°C, whereas the other three cultivars exhibited smaller fluctuations, with *HymCHS* expression being suppressed at -12°C (**Figure 4C**). *HymANS* demonstrated greater changes in expression in ‘Forever summer’ and ‘Magic coral’, peaking at -4°C and -8°C, respectively, whereas the other two cultivars exhibited minimal variation (**Figure 4D**).

## 4 Discussion

### 4.1 Effects of low-temperature stress on the physiology and biochemistry of hydrangea

MDA is a suitable indicator of the degree of stress-induced damage to plants, and it reflects the degree of cell membrane peroxidation. The higher the MDA content is, the more severe the damage to plant cell membranes (Megha et al., 2018; Wei et al., 2007). When the biofilm system is damaged, the semipermeability of the cytoplasmic membrane gradually decreases, and organic matter or salts seep out of cells, causing an imbalance in osmotic pressure inside and outside cells and an increase in relative conductivity (Mostofa et al., 2015). The results of this study revealed that the MDA content in the leaves of the four hydrangea cultivars increased overall with increasing low-temperature stress, indicating that as the temperature stress continued to decrease, the degree of damage to the biofilms gradually increased. Moreover, the lower the LT50 value of the cultivar ‘Magic coral’ was, the smaller the increase in the MDA content in its leaves. These findings are consistent with those of previous studies on plants such as *Elymus sibiricus* (Li et al., 2025) and *Nicotiana tabacum* (Song et al., 2024).

Under normal growth conditions, the production and clearance of ROS in plants occur in a dynamic equilibrium that avoids damaging plant cell membranes. However, under adverse conditions, this balance is often disrupted, causing the production of a large amount of ROS and resulting in the peroxidation of membrane lipids, and these processes ultimately lead to membrane system damage and cell oxidation (Cao et al., 2017; Gechev et al., 2003; Qian et al., 2024). Plants have endogenous antioxidant protection systems, such as those involving SOD, POD, and CAT, to protect themselves from ROS damage (Gechev et al., 2003; Van Camp et al., 1997; Wu et al., 2019). These systems can help clear excess ROS from the plant body, maintain the dynamic ROS balance, and reduce or alleviate stress-induced damage, thus increasing plant tolerance to adverse conditions (Feng et al., 2021; Li et al., 2021; Suzuki et al., 2012). The results of this study revealed that the SOD, POD, and CAT activity in the four hydrangea cultivars first increased but then decreased throughout the entire low-temperature stress process. During the early stage of low-temperature stress, the activities of SOD, POD, and CAT in the various hydrangea cultivars increased, indicating that low-temperature stress gradually increased the activity of SOD, POD, and CAT to protect the plants from damage. When the temperature continued to decrease, the activities of SOD, POD, and CAT decreased, indicating that at this time, low-temperature stress exceeded plant tolerance and damage to the plant body could have occurred. These findings are consistent with those of previous studies on plants such as tobacco (Gechev et al., 2003) and *Arabidopsis* (Cao et al., 2017).

Soluble sugars and soluble proteins are important osmoregulatory substances in plants. When plants are subjected to low-temperature stress, their soluble sugar and soluble protein contents increase to increase their osmotic pressure, increase their water retention capacity, maintain normal cell membrane function, and protect them from injury (Li et al., 2024a, 2024; Shahryar and Maali-Amiri, 2016). The results of this study revealed that under low-temperature stress conditions (-4°C), the soluble sugar and protein contents in hydrangea leaves were significantly greater than those in the control. At this time, the osmotic regulation ability of hydrangea leaves was enhanced, which may be one of the main mechanisms by which hydrangeas resist low-temperature stress.

### 4.2 Response of genes related to cold resistance in hydrangea under low-temperature stress conditions

Low temperature is one of the key environmental factors that influences plant growth and development, and plants can activate various defensive mechanisms to adapt to cold stress (Wang et al., 2016). Signal transduction mediated by transcription factors is one of the most rapid and effective defense responses, and it is an important component of complex regulatory networks in plants (Ashraf, 2010; Lee et al., 2005). Numerous studies have shown that ethylene response factor (ERF) transcription factors play crucial roles in shaping the responses of plants to various biotic and abiotic stresses (Mizoi et al., 2012; Pan et al., 2012; Ritonga et al., 2021). These genes can bind to cis-acting elements, such as the GCC box and DRE/CRT, to regulate the ethylene response and stress responses, such as responses to drought and low temperatures, respectively. The conserved sequence of the GCC box is AGCCGCC, which is located mainly in the promoter of alkaline PR genes, whereas the conserved sequence of DRE/CRT is CCGAC, which is found in many low temperature-responsive or drought-induced genes (Sakuma et al., 2002; Wu et al., 2022). In this study, an ERF gene in hydrangea was shown to play a regulatory role in the low temperature-induced defense process. Its expression first increased but then decreased with increasing low-temperature stress. The expression of this gene was high, and the degree of change was large in the cold-resistant ‘Forever summer’ and ‘Magic coral’ cultivars. In the future, we will continue to analyze the gene function of this transcription factor and explore its biological functions in depth.

Antioxidant enzyme-encoding genes include those that encode SOD, POD, CAT, and ascorbate peroxidase (APX). These genes regulate ROS levels by regulating corresponding antioxidant enzymes, thus protecting cells from oxidative damage (Salih et al., 2024). Under adverse stress conditions, plants directly or indirectly regulate the expression levels of antioxidant enzyme-encoding genes through hormone signals, transcription factors, ROS signaling molecules, etc., to adapt to environmental changes (Lin et al., 2024). In this study, *HymPOD*, which is an antioxidant enzyme-encoding gene, also exhibited a significant response to low-temperature stress, first increasing but then decreasing in response to ROS, and the greatest change was observed in ‘Forever summer’, which is highly consistent with the measured level of POD enzyme activity.

The formation and accumulation of secondary metabolites are closely related to temperature, and both high and low temperatures can affect the activity of secondary metabolite enzymes in plants. Low temperatures are more conducive to the generation of plant flavonoids (Bruňáková et al., 2022; Daryanavard et al., 2023). Anthocyanins, which are a type of flavonoid, also play important roles in adapting to cold stress (Heydari et al., 2023; Moradbeygi et al., 2020). Ye et al. (2025) reported that compared with wild-type tobacco, transgenic tomato *AN2* gene-overexpressing tobacco exhibited increased expression levels of several anthocyanin biosynthesis-related genes and a significantly increased anthocyanin content, thus increasing the resistance of transgenic tobacco to cold stress. Li et al. (2017) reported that the overexpression of the Arabidopsis *UGT79B2/B3* genes significantly increased plant tolerance to low temperatures. The expression of *UGT79B2* and *UGT79B3* is directly controlled by *CBF1* in response to low temperatures. These results indicate that *UGT79B2* and *UGT79B3* encode anthocyanin glycosyltransferases that are regulated by *CBF1* and increase plant tolerance to low temperatures by regulating anthocyanin accumulation. The overexpression of the *MdbHLH33* gene in apples can generate callus tissue of red meat, and the overexpression of the *MdMYB15L* gene in callus tissue can reduce the synthesis level of anthocyanins, thus reducing their ability to resist low temperatures. The *LBSMdMYB15L* gene is the binding site sequence of the *MdMYB15L* gene. The overexpression of the *LBSMdMYB15L* gene in red-fleshed callus tissue overexpressing the *Mdb-HLH33* gene can also reduce the ability of this organism to resist low temperatures. Therefore, the *MdMYB15L* gene plays an important role in shaping anthocyanin-induced resistance to low-temperature stress (Xu et al., 2018). Therefore, the introduction of structural or regulatory genes that are related to anthocyanin biosynthesis into plants can significantly increase the expression of anthocyanin biosynthesis-related genes, increase the accumulation of anthocyanin, and thus increase the resistance of transgenic plants to cold stress.

Analysis of the expression patterns of the four genes related to cold resistance in hydrangea provides reference values for the molecular breeding of cold-resistant clones of hydrangea. However, to analyze the molecular mechanisms underlying cold resistance in hydrangea comprehensively and in depth, increasing the expression patterns of more candidate genes related to cold resistance and performing functional analyses are necessary. Therefore, cloning full-length genes, constructing overexpression vectors for cold-resistant hydrangea functional genes, and establishing genetic transformation systems will be the focus of future work.

The analysis of the expression patterns of the four genes related to cold resistance in hydrangea provides reference values for the molecular breeding of cold-resistant clones of hydrangea. However, to analyze the molecular mechanisms underlying cold resistance in hydrangea comprehensively and in depth, it is necessary to increase the expression patterns of more candidate genes related to cold resistance and perform functional analysis. Therefore, cloning full-length genes, constructing overexpression vectors for cold-resistant hydrangea functional genes, and establishing genetic transformation systems will be the focus of future work.

### 4.3 Comprehensive evaluation of the cold resistance evaluation of the four hydrangea varieties

Low temperature is one of the main factors that limits plant growth and has adverse effects on various aspects of plant physiology. Owing to the complex mechanism underlying plant cold resistance, which involves the combined actions of multiple systems that are regulated by multiple specific numbers of cold resistance genes, it is difficult to fully and accurately reflect the magnitude of plant cold resistance on the basis of a single indicator alone (Lu et al., 2022; Qi et al., 2025; Zhang et al., 2025). Therefore, a combination of multiple indicators is typically needed to comprehensively evaluated plant cold resistance. Currently, the commonly used methods for the comprehensive evaluation and analysis of plant cold resistance include cluster analysis and membership function analysis methods (Lu et al., 2022; Zhang et al., 2025), with the latter being the method that is most commonly used. In this study, to accurately assess the cold resistance of these cultivars, we also adopted membership function analysis to comprehensively evaluate the cold resistance of the four hydrangea cultivars. On the basis of individual analyses of the responses of seven physiological and biochemical indicators (such as leaf REC, MDA content, SOD activity, POD activity, CAT activity, soluble sugar content, and soluble protein content) under low-temperature stress conditions, the LT50 of hydrangeas was calculated by combining REC with a logistic regression equation. Combined with the anatomical structural characteristics of leaves and the expression of cold resistance-related genes, a comprehensive evaluation of the cold resistance of the four hydrangea cultivars was conducted. The analysis revealed that the conclusions derived from the anatomical structure of the leaves were essentially consistent with those derived from the physiological indicators and LT50 values. The order of cold resistance of the four hydrangea cultivars was as follows: ‘Forever summer’ > ‘Magic coral’ > ‘Tabei’ > ‘Huabao’. Therefore, in the future, ‘Forever summer’ and ‘Magic coral’ can be selected as the main hydrangea cultivars for landscape construction in cold northern regions.

## 5 Conclusions

In this study, four hydrangea cultivars were used as research materials to comprehensively evaluate cold resistance and screen suitable cultivars. The results revealed significant differences in the leaf anatomical structure among the four cultivars, especially in terms of leaf thickness, palisade tissue thickness, and sponge tissue thickness, providing a structural foundation for assessing differences in cold resistance among the four cultivars. Under low-temperature stress conditions, with decreasing temperature, the relative conductivity of the leaves of the four hydrangea cultivars continuously increased, with the LT50 ranging from -2.404°C to -3.133°C. Notably, lower values were observed in ‘Magic coral’ and ‘Forever summer’. Under low-temperature stress conditions, the rate of MDA production in leaves significantly increased, with a greater degree of increase observed in ‘Huabao’. The levels of antioxidant enzyme activity and soluble substances in hydrangea leaves first increased but then decreased with decreasing temperature, especially in ‘Magic coral’ and ‘Forever summer’. The expression levels of the *ERF*, *POD*, *CHS*, and *ANS* genes, which are related to cold resistance, also first increased but then decreased with decreasing temperature, especially in ‘Forever summer’ and ‘Magic coral’. The low temperature-induced upregulation of antioxidant enzymes and cold resistance-related genes enhances intracellular antioxidant defense, helps maintain cell membrane stability in hydrangea, and thus increases cold resistance. On the basis of the abovementioned indicators and using the membership function method for a comprehensive evaluation of the cold resistance of the four hydrangea cultivars, the results revealed that the cold resistance of ‘Forever summer’ and ‘Magic coral’ was greater than that of ‘Tabei’ and ‘Huabao’. These research results provide a theoretical basis for the introduction, domestication, and landscaping application of hydrangeas in cold northern regions.

## Data Availability

The original contributions presented in the study are included in the article/supplementary material. Further inquiries can be directed to the corresponding authors.
